# Patient Management Strategies in Perioperative, Intraoperative, and Postoperative Period in Breast Reconstruction With DIEP-Flap: Clinical Recommendations

**DOI:** 10.3389/fsurg.2022.729181

**Published:** 2022-02-15

**Authors:** Kuo Chen, Narasimha M. Beeraka, Mikhail Y. Sinelnikov, Jin Zhang, Dajiang Song, Yuanting Gu, Jingruo Li, I. V. Reshetov, O. I. Startseva, Junqi Liu, Ruitai Fan, Pengwei Lu

**Affiliations:** ^1^Department of Breast Surgery, The First Affiliated Hospital of Zhengzhou University, Zhengzhou, China; ^2^Ministry of Health of the Russian Federation, Sechenov University, Moscow, Russia; ^3^Department of Radiation Oncology, Cancer Center, The First Affiliated Hospital of Zhengzhou University, Zhengzhou, China; ^4^Department of Oncology Plastic Surgery, Hunan Province Cancer Hospital and the Affiliated Cancer Hospital of Xiangya School of Medicine, Central South University, Changsha, China; ^5^L.L. Levshin Institute of Cluster Oncology, Moscow, Russia; ^6^Academy of Postgraduate Education, The Federal State Budgetary Unit FSCC, Federal Medical Biological Agency, Moscow, Russia; ^7^Cancer Center, The First Affiliated Hospital of Zhengzhou University, Zhengzhou, China

**Keywords:** breast reconstruction, DIEP flap, surgery, patient management, perioperative, intraoperative, postoperative period

## Abstract

**Background and Objective:**

Deep Inferior Epigastric Perforator (DIEP) flap is a tissue isolated from the skin and subcutaneous tissue of the lower abdomen or rectus muscle to foster breast reconstruction. There is limited information about DIEP-flap induced complications associated with breast reconstruction surgery.

**Evidence:**

We conducted a systematic review of the published literature in the field of breast cancer reconstruction surgery. Information was gathered through internet resources such as PubMed, Medline, eMedicine, NLM, and ReleMed etc. The following key phrases were used for effective literature collection: “DIEP flap”, “Breast reconstruction”, “Patient management”, “Postoperative DIEP”, “Intraoperative anticoagulant therapy”, “Clinical recommendations”. A total of 106 research papers were retrieved pertaining to this systematic review.

**Conclusion:**

A successful breast reconstruction with DIEP-flap without complications is the priority achievement for this surgical procedure. This study provides various evidence-based recommendations on patient management in the perioperative, intraoperative, and postoperative periods. The clinical recommendations provided in this review can benefit surgeons to execute breast reconstruction surgery with minimal postoperative complications. These recommendations are beneficial to improve clinical outcomes when performing surgery by minimizing complications in perioperative, intraoperative, and postoperative period.

## Highlights

- Breast reconstruction with the DIEP-flap can be surgical choice in the cases of structural restoration of the anterior chest wall anatomy after a mastectomy.
- This is a systematic review with evidence-based recommendations on patient management in the perioperative period after DIEP flap breast reconstruction.
- This study represents surgery an important stage in complete functional, emotional, psycho-social, and aesthetic patient rehabilitation with improved quality of life after radical mastectomy.
- The study recommendations have a strong potential to improve clinical results when performing breast reconstruction with the DIEP-flap, and will serve as a basis for new prospective studies on topics covered in this study.

## Introduction

Breast reconstruction is a significant approach in the patients who received radical mastectomy to improve their quality of life by minimizing psychosocial stress. This kind of strategy is a crucial element in the successful therapy and management of breast cancer (1, 2). For instance, the autologous microvascular breast reconstruction reported to produce typically significant clinical outcomes; and this kind of reconstruction can be performed using DIEP flap or a muscle-sparing (MS) free “transverse rectus abdominis musculocutaneous” (TRAM) flap (3, 4). However, there are several perioperative, intraoperative, and postoperative complications reported during DIEP-flap breast reconstruction surgery but the clinical information pertaining to the management of these complications in surgical oncology are minimal. For instance, DIEP-flap complications can be practically separated into two groups: first group- complications associated with technical difficulties in flap mobilization such as anastomosis formation, perforate vessel traumatization, surgical mistakes in vascular anastomosis completion (5, 6); a second group - complications due to the mistakes in the patient preparation and management (7). Based on the classification of complications, we have performed a systematic review of literature in regard to the most important aspects of preoperative, intraoperative, and postoperative patient management when performing a DIEP-flap breast reconstruction.

The current review outlines articles, clinical cases, different publications pertinent to the quality of care and clinical recommendations of patient management when performing autologous breast reconstruction using a DIEP-flap. Furthermore, this review delineates the proper patient management in the preoperative period forms significant clinical recommendations on intraoperative management, and defines an appropriate postoperative management protocol suggesting a viable strategy for the breast reconstruction to foster patient management effectively in the preoperative, intraoperative, and early postoperative periods.

### Overview

The usage of autologous tissue isolated from the lower abdominal wall is a reliable and popular method of breast reconstruction. The technique was first described by Holmstrom in 1979 and popularized by Hartrampf et al. in 1982 (8). The history and development of modern reconstructive surgery reported as a justified strategy to improve operative technique, consequently patient management in the perioperative period in order to minimize the postoperative complications. Substantial breakthrough in reconstructive and plastic surgery was seen in 1989, when a prospective surgeon G. Ian Taylor formulated the angiosome theory of vascularization (9).

The constant commitment of surgeons to refrain from executing the traumatizing surgical operations began due to the new era of developments in the perforator flap-based reconstruction surgery (4). A new stage in the development of breast reconstructive surgery was the DIEP-flap based reconstruction technique, which could allow minimal anterior abdominal wall traumatization, and sparing the rectus abdominis muscle and minimal injury of the abdominal aponeurosis (3). This new era of breast reconstructive surgery was dominated by the DIEP-flap. Blondeel P. N. et al. showed that compared to the TRAM-flap, mobilization of the DIEP-flap significantly can decrease surgical traumatization of the rectus and oblique abdominis muscles, which minimize the incidence of weakness in the anterior abdominal wall during the postoperative period (3). Despite the widely accepted advantages of minimally traumatizing techniques over other intervention methods, it does not prevent complications completely (9). In addition, the attributes of new surgical methods in breast reconstruction can increase the specter of possible complications (3, 7). DIEP-flap is currently referred to as the gold standard in reconstructive mammoplasty. The usage of DIEP-flap allows for minimization of donor site morbidity (3, 10–13), but the reconstructive procedure still remains a long and strenuous surgery, with a median operative time of 6–7 h.

## Methods

### Data Sources

The literature for this systematic review was gathered through internet resources, such as PubMed, Medline and others (eMedicine, NLM, ReleMed). All the data acquired for this study were completely in accordance with the guidelines outlined in the PRISMA statement. An automatic search with manual sorting of the selected articles was performed. The following key phrases were used: “DIEP-flap”, “breast reconstruction”, “patient management”, “postoperative DIEP”, “intraoperative anticoagulant therapy”, “clinical recommendations”. A total of 106 papers were retrieved relevant to our research and subjected for primary evaluation. Total 56 significantly informative papers were selected after primary screening procedures, and were categorized according to sources including pubmed, medline, others.

### Study Selection

**Figure 1:**
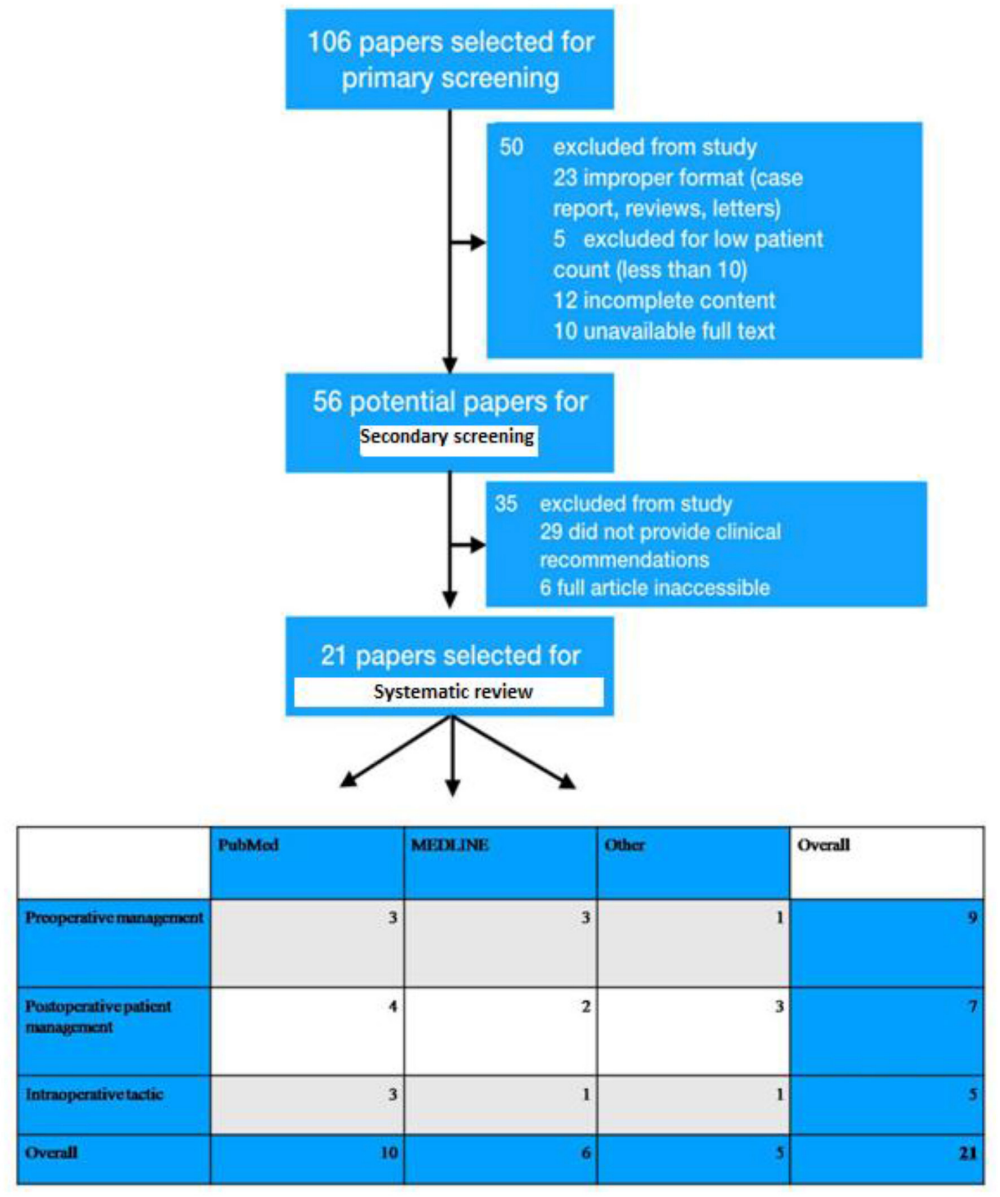
Depiction of exclusion and inclusion criteria of patients who were undergone breast reconstruction and total 106 papers were primarily screened with respect to actuality, publication date, access to article text, content, number of patients, and complete flap-loss rate. Primary screening was executed and selected 56 research papers for secondary screening. Consequently, a two-stage screening process was performed and selected 21 research papers for this study.

Potentially acceptable articles for systematic review were considered, which deciphered the topic for breast reconstruction with free flaps, including DIEP-flaps, and the analysis of this article information gave specific recommendations on patient management. Total 106 papers were primarily screened with respect to actuality, publication date, access to article text, content, number of patients, and complete flap-loss rate, whereas 56 research papers were chosen for secondary screening. Following a two-stage screening process, 21 research papers were selected for further study (Figure 1).

### Data Extraction

**Figure 2:**
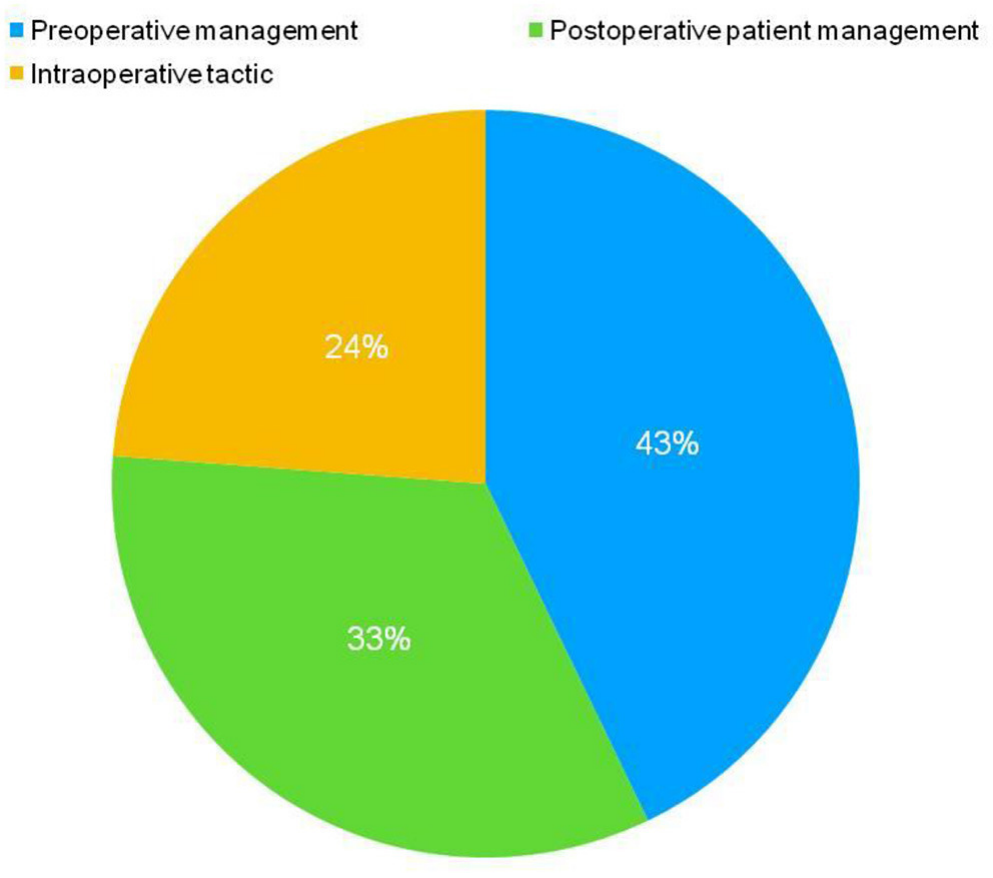
Three main groups were chosen for proper categorization for further inferences to delineate the efficient patient management in preoperative period during DIEP flap-based breast reconstruction. This categorization was performed according to the preoperative preparation, postoperative patient management, and choice of the intraoperative therapy.

According to international standards provided by the “systematic review of observational Studies in epidemiology”, the selection and categorization of data was performed according to the following criteria: number of patients included in the study, amount of flap transfers performed, incidence of complications, type of surgical intervention, patient age, year and type of publication, recommendations on patient management. Three main groups for proper categorization were derived as per the preoperative preparation, postoperative patient management, and choice of intraoperative therapy (Figure 2). The results of these three separate categories were reviewed and analyzed separately. Systematically, the crucial inferences were addressed in this review which might be beneficial to surgeons during the patient management during or after the breast reconstruction with DIEP flaps.

## Results

### Preoperative Period

The preoperative period was overviewed by ascertaining the total 9 clinical studies in order to describe the effective patient management. A literature review of these studies outlining clinical recommendations on patient management was delineated in the preoperative period mainly in the patients undergoing breast reconstruction with a DIEP-flap.

According to previous studies, patient preparation is one of the most important factors, which could influence postoperative rehabilitation and incidence of complications (14–16). Clinical manifestations of postoperative complications are dependent on the quality of preoperative patient preparation. A plethora of clincial reports deciphered the correlation between low perioperative morbidity rates and proper “preoperative patient preparation” for the surgical treatment and postoperative rehabilitation. Hence, preoperative preparation should ascertain the “anatomical and morphological aspects of the donor and recipient sites” (7, 17–19). The success of a DIEP flap-based breast reconstruction depends on several factors; For instance, the perfusion of transferred flap is the most important factor that determines postoperative complications. Standards of perfusion monitoring of DIEP-flap should be carried out by observing temperature, vascular pulse, vessel diameter correlation, capillary response prior to surgical intervention (7, 14, 20–22). To decrease the overall complication rate, it is necessary to understand the “dynamics and anatomy of blood flow” through the deep and superior inferior epigastric systems (7). In case of dominant flap perfusion through the superficial inferior epigastric system (SIEA system), the dissecting SIEA artery during flap mobilization can increase the risk of partial DIEP-flap necrosis (23). The number of perforators available for mobilization plays a crucial role in flap viability. Comparatively, decline in the incidence of complications was observed in breast reconstructions with the DIEP-flaps based on two or more perforators used for anastomosis, as well as two and more veins (22).

Minqiang et al. have reported 22 cases of breast reconstruction with DIEP-flap. The preoperative visualization in these patients was conducted via a multidetector spiral computed tomographic angiography (MSCT-angiography). The study showed minimal complication rates including 0% of severe complications, and less than 5% postoperative complications with light and medium severity (24). Innovations in the preoperative patient visualization techniques and protocols prior to breast reconstruction with the DIEP-flap are associated with a lower overall operative risk, as well as a lower rate of perioperative complications. Preoperative CT-angiography can minimize operative time, less anterior abdominal wall morbidity subsequently enhance the conditions for a surgical intervention. Recently, CT-visualization is used not only to assess the quality, quantity, and functionality of the perforator vessels, but also to assess the suitability of flap tissue, which is available for mobilization (25). The algorithm “Volumetric Planning” reported by Chang & Ooi showed significantly impressive results in the flap preparation and preoperative availability for assessing mobilization flap volume prior to surgical procedure. The incidence of postoperative complications is statistically less in case of proper preoperative flap perfusion zone analysis (26, 27). We recommend performing a thorough preoperative visualization, mapping, and volume assessment to achieve the best postoperative results during DIEP flap-based breast reconstruction.

A significant amount of observational studies performed by reconstructive and plastic surgeons have concluded that adequate preoperative planning is an inseparable part of a successful surgical intervention to promote the complication-free postoperative period (25, 28). Different examination methods are recommended by different authors including CT-visualization, MRI-angiography, pPCF, and ultrasound-dopplerography (29–31).

Adequate preoperative patient preparation includes the evaluation and modification of risk factors. Previous observational studies have examined a total 758 cases of breast reconstruction with the DIEP-flaps, and underlined the importance of the evaluation of potential risk factors prior to the surgical intervention. The main etiopathogenetic factor in the manifestation of postoperative complications after a successful surgical reconstruction is “obesity”. A high BMI is one of the significant factors which can confer to a higher incidence of complication rate in both the donor and recipient sites (14). Despite this fact, the DIEP-flap is still the method of choice, because this kind of procedure has been associated with a statistically low incidence of complications in the patients undergoing breast reconstruction, when compared to other reconstruction methods (21). Nahabedian et al. showed that smoking and patient age are not the major risk factors in the pathogenesis of partial necrosis and venous thrombosis after the breast reconstruction; the main cause of these complications is overweight in these patients (32). Even though there are several International Society of Aesthetic Plastic Surgery (ISAPS) recommendations pertinent to the importance of smoking refusal at least two weeks prior to surgery, there are no statistically significant reports describing the “loss of DIEP-flap, microvascular complications or partial fat necrosis” in the patients with smoking and underwent breast reconstruction. On the other hand, the smoking patients exhibit a higher incidence of postoperative abdominal weakness and infection. Patients with a “pack/year index of 10 and more” are considered to be at the high risk of gaining postoperative complications. Surgery is not recommended for these patients (33). In their observational study, Selber et al. showed a correlation between certain risk factors and complication occurrence. For instance, the prior radiation therapy was consistent with seroma formation in the postoperative period in DIEP-flap based breast reconstruction (34).

Hormonal and chemotherapy prior to the surgical intervention of breast reconstruction with the DIEP-flap could invoke a higher incidence of vascular complications in the patient groups (35). It is a proven fact that the administration of tamoxifen prior to the surgery typically has a significant impact in inducing microvascular complications. Therefore, it is recommended to avoid administration of tamoxifen 14 days prior to the surgical intervention, and resume the administration of this drug after surgery (36).

### Intraoperative Tactic

There were a total of 5 studies that overviewed 937 flaps and these reports can ascertain the intraoperative breast reconstruction and patient management. The mean complication rate was 2.9%, and mean complete flap loss rate was 1.2%. A literature review has been performed as a secondary procedure to ascertain the patient management strategies in the intraoperative period during breast reconstruction with DIEP-flap based surgical intervention.

Temperature control is another significant factor in the patient management during DIEP flap-based surgery. Previous studies reported a statistically significant correlation between “hypothermia” with a higher risk of incidence of postoperative complications (37, 38). Intraoperatively, the patients are subdued to longer periods of exposition, which predetermines hypothermia. Moreover, the anesthesia-related interventions can influence the thermoregulatory mechanisms of the patient (37, 38). For instance, the increased risk of intraoperative hypothermia could cause a higher risk of intraoperative complications; therefore it is another significant strategy to control the intraoperative patient temperature during the surgical intervention of breast reconstruction and the patient temperature should not be less than 35°C. During flap mobilization, it is recommended to monitor the patient's temperature and persistently maintained 37°C (37).

A previous study analyzed the adequacy of different intraoperative anticoagulation regimen during surgical intervention of breast reconstruction (39). This study reported that adequate heparin therapy with low-molecular-weight heparin (LMWH) is necessary as a precaution to prevent vascular thrombosis. However, the intraoperative heparin administration did not show any statistically significant influence on the microvascular anastomosis competence (level of evidence 2b). The significance of LMWH administration at the time of postoperative period is to minimize the vascular complications after breast reconstruction. Furthermore, the administration of dextran is strictly prohibited due to the complete flap loss as observed in these studies (40).

Fibrin glue usage during the surgical intervention has significant implications as a stabilizing agent to minimize the incidence of perioperative complications, and also reduce the rate of complete flap loss. Only 0.9% of complete flap loss was observed out of the total 301 transferred flaps. Fibrin glue is useful as a microvascular anastomosis stabilizing agent and it allows a significant decrease in the perfusion and microvascular-related complications, consequently improving the overall flap viability (41).

Enajat et al. delineated the Swedish-Australian microsurgical intervention of 564 cases of breast reconstruction with the DIEP flaps and suggested several useful conclusions for executing the proper intraoperative technique for patient management (42). This study recommended the completion of two venous anastomoses per flap instead of just one, because this kind of approach can minimize the risk of acquiring venous complications. In order to reduce the incidence of venous complications during breast reconstruction with the DIEP-flap, it has been recommended performing “venous superdrainage” during surgery. The significant goal of this method is to provide a back-up drainage system via a secondary venous anastomosis between the “superficial flap venous system” and a “recipient vein” (thoracodorsal vein, lateral pectoralis vein, intercostal vein, medial subcutaneous vein). The rate of venous congestion in cases of performing a venous superdrainage is significantly lower when compared to the patient cases without a secondary venous outflow substrate (43).

The prevalence of fat necrosis in the recipient zone is another postoperative complication elicited due to the variability in the diameter of the anastomosed vessels after breast reconstruction with DIEP flap (43–46). Thus, the importance of vessel diameter conformity in the reduction of postoperative complications has been observed by several authors (41, 44–47). Furthermore, the donor site complications can play a significant role in the postoperative morbidity after the breast reconstruction with DIEP-flap (48). In order to avoid donor site complications, it is recommended to follow certain sequential actions during surgical intervention. For instance, the intraoperative direction of muscular nerve branches innervating the rectus abdominis muscle can facilitate their preservation, which further helps to minimize postoperative morbidity of the donor site. A significant risk factor of anterior abdominal wall weakness in the postoperative period is intraoperative bilateral mobilization of the DIEP-flap (32, 49).

A few prospective randomized clinical studies reported a correlation between quality and quantity of infusion and the risk of developing postoperative complications (50–52). They analyzed 354 cases of microsurgical breast reconstruction with strictly regulated infusion tactics. Only 0.8% of complete flap loss was observed and minimal complication rate also observed which was due to the implementation of an optimal infusion tactic i.e. crystalloid infusion rate between 3.5 ml−6 ml/kg per hour in the first 24-h postoperative period (level of evidence 2b) (41, 53). Crystalloid infusion should not exceed 130 ml/kg in the first 24-h postoperative period. Infusion volume in the intraoperative period is derived from overall biological fluid loss, a parameter which should be counted for the levels of intravenous infusion (54).

Previous studies deciphered the intraoperative evaluation of perfusion dynamics in the revascularized flap (55, 56). The instrumental intraoperative visualization of flap perfusion, as a technique could help to evaluate flap viability as well as to predict the negative impact of zones with reduced tissue perfusion. Application of the intraoperative dynamic infrared tomography (DIRT) reported typically fruitful results during the assessment of quality of flap perfusion (57). The quality of flap perfusion defines the consequent surgical tactic, including, but not limited to flap volume correction. Intraoperative thermography can significantly minimize the risk of postoperative perfusion-related complications (58, 59). In addition, laser-assisted angiography with indocyanine green is another method applied to evaluate the intraoperative flap perfusion. This method is used to visualize vessel anatomy in the preoperative period, and also to evaluate perfusion dynamics of the transferred free flap, as well as quality and stability of the microvascular anastomosis (56, 60).

Furthermore, it is crucial to evaluate the risk of developing postoperative complications in patients, who received a blood transfusion during surgery. The intraoperative transfusion is required directly based on the length of surgery and volume of reconstructive intervention. Appleton et al. recorded a higher complication risk in the patients with bilateral breast reconstruction with DIEP-flap, as well as in patients with a prolonged operative time, who received hemotransfusion (61).

### Postoperative Period

In order to adequately analyze the proper patient management tactic in the postoperative period, a total of 3,335 cases of breast reconstruction with the DIEP-flap were included in the study. The mean complication rate in the selected cases was 4.44% whereas the “severe complications rate”, and “complete flap loss rate” were 3.73, and 2.48% respectively.

In addition, adequate pain management is an inseparable part of postoperative treatment. Patients undergoing the breast reconstruction using DIEP-flap can be segregated into two different categories based on the pain threshold: “patients with a normal pain threshold', and ‘patients with a low pain threshold”. The first group (70–75%) requires patient-controlled treatment (PCT) for mitigating pain in the initial two days after surgery followed by the administration of oral analgesics (62, 63). The second patient group (25–30%) consists of patients, who need a longer course of PCT- upto three days; this group is characterized by a longer hospitalization stay and a lengthier rehabilitation period. Furthermore, the patients after a simultaneous mastectomy with breast reconstruction more often fall into the second patient group (62). In order to minimize the requirement of narcotic analgesics administration, the authors recommend different pain management strategies postoperatively after breast reconstruction (63). Blockade of the transverse abdominal space with the ultrasound control can eliminate the requirement of narcotic analgesic administration. Catheterization of the donor zone for local anesthetic administration can allow quality anesthesia for up to 72 h without the need of narcotic analgesics (64). Epidural anesthesia is another significant pain control strategy during surgery, which allows adequate pain management without the intravenous administration and PCT. As per several studies, the most innovative anesthetic methods are local anesthesia applied during blockade and catheterization (65, 66).

The loss of hemoglobin is one of the most important complications in postoperative patient management. The length of hospitalization, overall blood loss, and blood transfusions considerably exacerbate the complications in postoperative period. Hemoglobin level is one of the most important indicators, which is an indicator of the hemodynamic and rheologic balance of the blood (67). A direct correlation was reported between anemia and the rate of postoperative complications during autologous breast reconstruction. This report also delineated a significant risk of developing postoperative complications observed in the patients with hemoglobin levels less than 100 g/l, which is not yet considered as an anemic state (67). Despite this, the authors conclude that hemoglobin levels less than 100 g/l, bilateral breast reconstruction, simultaneous reconstruction, and blood transfusion are statistically significant and indicate postoperative complication development (68). The length of surgical intervention defines the loss of hemoglobin. For instance, one hour of surgery is accompanied by an average loss of 0.25 g/l of hemoglobin. Intraoperative complications conferred substantial rise in the average loss of 0.45 g/l hemoglobin. Each gram of the removed tissue amounts to a corresponding average of loss of 0.001 g/l hemoglobin. For instance, tranexam (TXM) is an effective drug, which prevents significant hemoglobin loss in the postoperative period. TXM administration could mitigate the average blood loss by 18.2 ml/kg (*p* = 0.001); therefore this drug can significantly be reported to increase flap survival prognosis (69). The influence of TXM administration on microvascular anastomosis was emphasized by Zhang and Wieslander. As per this study, the administration of a clinical dose and double dose of tranexam (14 mg/kg and 28 mg/kg accordingly) has not exhibited a statistically significant effect on thrombus formation and bleeding in microvascular anastomosis (70).

**Fluid loss compensation** is primarily performed according to volumetric parameters. Total 354 cases of breast reconstruction with free abdominal flaps were examined; these reports showed the optimal rate of infusion parameters for the perioperative period (51, 52). The optimal measure of **crystalloid infusion** should be between 3.5 ml-6 ml/kg per hour to replenish the fluid loss. Blood transfusion should be performed according to clinical readings and if the patient's hemoglobin levels are less than 70 g/l (level of evidence 2b). Crystalloid infusion rate should not exceed 130 ml/kg a day. Existing data does not support the usage of albumin over synthetic colloid solutions (52). Proper strategies of fluid infusion performed in this study to reduce the overall complication rate to 4.1%, where the complete flap loss only 0.8%.

Maintaining flap perfusion is another significant surgical priority after breast reconstruction. Perfusion control and pharmacological support are necessary in order to maintain flap perfusion effectively. Flap perfusion control is performed using dynamic infrared thermography (DIRP). This strategy allows surgeons to detect perfusion pathology at the earliest; therefore timely management of complications can be executed. Early hypoperfusion identification could allow a conservative approach in preventing further development of serious complications, such as border necrosis, wound dehiscence, adipose necrosis, and flap loss (71). Standard strategies such as angiography and ultrasound dopplerography (USDG) could be used to evaluate the anastomosed vessels pertaining to the quality of flap perfusion, which can also be analyzed by subjective methods. The application of Doppler-catheter “Cook-Swartz” implantation has shown a significant decline in false-diagnosis of complications and allowed surgeons to detect early microvascular complications. This method is referred to as a new method, which could be useful to predict and prevent microvascular complications by ascertaining the tissue oxygen saturation (StO2, ΔStO2), and saturation change speed (ΔStO2/Δt). Rate of change of ΔStO2/Δt by −20% in 30 min preceded vascular complication manifestation (72, 73). This kind of monitoring can be executed with the aid of a “T.Ox Tissue Oximeter” and it can predict the development of vascular complications 60 min prior to their true manifestation (74).

The flap perfusion quality through the pharmacological therapies is an important aspect for the effective postoperative patient management. Previous studies deciphered the effectiveness of heparin medication in the postoperative period. Among 493 cases of free flap reconstruction, a higher risk of complication development was observed in the patient cases receiving inadequate administration of heparin therapy. Postoperative subcutaneous administration of heparin significantly can mitigate the risk of microvascular thrombosis. There is no statistically significant data in favor of systemic administration of heparin when performing free flap transfer (level of evidence 2b). The administration of dextran as a prophylaxis for venous thrombosis is contraindicated due to a higher incidence of microvascular and perfusion-related complications when using this drug (level of evidence 1b) (39, 75).

The need for radiation therapy after a mastectomy and breast reconstruction has been reported to be varied between 25–30% (76). A majority of surgeons are very concerned to use radiation therapy onto the “area of anastomosis” as the radiation could enhance the development of associated complications. Chatterjee et al. conducted a clinical study, which analyzed the rate of flap volume loss depending on dosage of radiation. The results of these studies showed that there is no statistically significant correlation between radiation therapy on the flap and flap volume loss (77). It is therefore not recommended to postpone radiation therapy. Above all the complications during DIEP flap-breast reconstruction were deciphered vividly in the following (Table 1).

**Table 1:** The patient management strategies in preoperative, intraoperaitve, and postoperative periods in order to minimize different complications observed in the DIEP flap-based breast reconstruction.

Modifiable risk factors	Diagnosis of modifiable risk factors, such as smoking, obesity, pharmacological therapy, hypertension and their correction.
Preoperative visualization	Rainbow 3D DIS, MSCT with angiography, Static CT-scanning, flap volume planning (calculation), perfusion zone analysis, choke-anastomosis analysis, Doppler ultrasound of recipient vessels.
Patient history	Obstetric history (previous births), scarring on the chest/abdomen, hernia formation data, operative intervention history, prior radiation, and chemotherapy, risk factor analysis.
Analgesic therapy	Preoperative administration of Gabapentin decreases pain and vomit postoperatively. Pump-anesthesia system implantation is necessary to reduce morbid sensation in the donor site. Gabapentin preoperatively. Ketorolak postoperatively.
Patient body temperature	Mean body temperature should be maintained at 37°C intraoperatively Core body temperature of less than 35°C is associated with a high complication rate
Anesthesia	Secondary epidural anesthesia decreases postoperative complication incidence. Sevoflurane protest vascular endothelium, better than propofol influences the capillary filtration index.
Infusion	Crystalloid infusion should be 3.5–6 ml/kg/h in the nearest postoperative period (24 h). Crystalloid infusion should not be more than 130 ml/kg/day (>5.4 ml/kg/h). Intraoperative infusion should not be more than 7 liters. Preoperative evaluation of Hct and Hgb levels is necessary. Hct levels of <30%, Hgb <100 g/l correlate with a higher complication rate. Blood transfusion should be performed only when Hgb is <70 g/l.
Spasmolytics	Persistent vasospasm can be corrected with topical administration of 4% lidocaine or papaverine. Application of these topical spasmolytic drugs correlates with a lower complication rate.
Vasopressors	Vasopressors are used to correct hypotensive conditions, and do not increase the complication rate. The cumulative effect of vasopressors does not influence the rate of complications. Dobutamine and dopamine improve cardiac output and arterial blood pressure, dobutamine also improves blood flow in the anastomosed vessels.
Anticoagulation therapy	Aspirin and subcutaneous heparin administration are recommended for thrombotic complication prophylaxis. There is no statistically significant data of complication manifestation in anticoagulant use (except for lower extremity venous thrombosis incidence). Intraoperative administration of systemic heparin does not influence the complication rate. Dextran is contraindicated: it increases overall complication rates, significantly increases overall flap loss incidence.
Analgesic	Anesthetic pump in the donor area significantly reduces pain in the postoperative period.
Surgical technique	Venous superdrainage is a recommended operative technique. Decreasing wound exposition, two working brigades in the donor and recipient cites is recommended. Fibrin glue sealant can improve anastomosis stability.
Perfusion control	USDG (Doppler ultrasound), DIRP, T.Ox Tissue Oximeter.

## Discussion

In this study, we reviewed a total of 6,475 cases of reconstructive interventions, covered in 21 studies. The mean complication rate in these studies on patient management in breast reconstructive surgery was 5.08% (78).

Patient body temperature should be properly monitored at the time of surgical intervention. This physiological parameter is often disregarded in the operating room. There is a proven correlation between hypothermia and the increased risk of complications. Patients during surgery are prone to the prolonged exposition, which predisposes them to hypothermia. Anesthesia administration could have a direct effect on the patient's thermoregulatory mechanisms, and the administration of anesthesia can modulate heat emission and heat production during surgery. Therefore, persistent control of the core body temperature should be implemented. As per the analysis of all the above reports systematically, we recommend the maintenance of a patient's core body temperature more than 35°C at all times during DIEP flap-based breast reconstruction. A mean temperature of over >37°C is recommended during flap transfer and vascular anastomosis. The mean recommended core body temperature in the operating room to prevent hypothermia should be 24°C. In all cases, it is recommended to warm patients prior to surgical intervention and 24–48 h postoperatively, preventing difference between peripheral and core body temperature of more than >2°C (54) (Table 2).

**Table 2:** Different clinical reports of overall complication rate (light/medium/severe) in the patient management in the DIEP flap-based breast reconstruction.

**Authors**	**Procedures/conclusions**	**Overall transferred flaps**	**Light complications**			**Medium severity complications**				**Severe complications**			**Overall complication rate**
			**Border necrosis**	**Transitory and reversible perfusion complications**	**Mild wound dehiscence**	**Spreading necrotic processes**	**Partial flap loss (<50%)**	**Donor site complications**	**Reversible complications (seroma, hematoma, infection, other)**	**Flap loss more than 50%**	**Complete flap loss**	**Other severe complications (including revision)**	
**Preoperative management**													
Nahabedian et al. (32)	Patient weight assessed prior to surgery, flap volume assessed and estimated, Rainbow 3D Digital Imaging System utilized.	20	0%	0%	5%	10%	NA	0%	15%	NA	5%	NA	5%
Gill et al. (14)	Risk factors are assessed. Risk factors are modified prior to surgery: smoking, hypertension, radiation therapy. Radiation therapy after a mastectomy is avoided.	758	12.9%	4.3%	20.2%	0.7%	2.5%	14.3%	5.9%	NA	0.5%	5.9%	7.46%
Minqiang et al. (24)	MSCT-angiography and preoperative imaging are performed and show effective reduction in postoperative complication rate.	22	5%							NA	0%	0%	1.6%
Santanelli et al. (79)	Obstetric anamnesis (number of births) is assessed. It is proved to be a significant factor in perfusion related complications of the flap.	287	12.9%		NA	NA	SM	NA	NA	SM	SM	NA	6.9%
Guerra et al. (18)	Risk factors and other criteria are noted to be precursors of complication development: smoking, obesity, age, radiation therapy, flap volume	280	12.5%	2.5%	NA		1.1%	2.1%	1.1%	SM	NA	6.4%	4.28%
O'Connor et al. (25)	Preoperative markup with prior quality vessel visualization is performed via dynamic CT visualization and/or static CT-scanning. Adequate preoperative markup allows for best flap volume transfer results.	632	6.9%			0.9%				0.31%	Consistent with findings of other authors	NA	2.52%
Ooi et al. (26)	Preoperative planning of flap volume is performed. The incidence of postoperative complications is significantly less in cases of preoperative assessment of flap perfusion zones.	Review of 5 different cohort	Consistent data with findings of other papers - author's statement.							NA	0.75%	NA	est. 4%
Parrett et al. (80)	Preoperative scar tissue is assessed and marked. It is necessary to understand donor site scarring and its role in flap perfusion. Scar tissue is excluded from the flap.	104	14%	NA	12%	NA	1%	5.1%	10.2%	NA	2.9%	NA	8.02%
**Intraoperative tactic**													
Bonde et al. (81)	Fast track surgery and reduction of LOS (length of hospital stay). Perioperative patient management. Less operative time correlates with less post-anesthesia consequences, less blood-loss and better results overall.	177	6.5%						9%	NA	2%	NA	5.83%
Enajat et al. (42)	Perform two venous anastomoses instead of one. This provides a lesser incidence of venous and perfusion complications, which provides better operative results.	291	SM	0%	SM	Similar results				SM	0%	SM	7.8%
Andree et al. (41)	Fibrin glue is used. This allowed for a reduction in overall complication rate and minimization of complete flap loss.	201	NA								0.9%	SM	NA
Lemaine et al. (82)	Heparin therapy by low-molecular weight heparin is performed to assess intraoperative vascular thrombosis risk. Anticoagulation therapy is recommended to oncologic patients on life-time courses.	56	0%	3.4%	NA	NA	1.7%	NA	5.9%	1.7%	1.9%	3.4%	2.57%
Liu et al. (83)	Temperature control. Hypothermia of 36,0-36,5C correlates with lower levels of microvascular complications.	212	Low flap thrombosis rate										NA
**Postoperative period**													
Chiu et al. (84)	Gabapentin is used as a preoperative drug. Postoperatively correlated with less pain. Pain management is performed.	25	Postoperative pain management.										NA
Zhong et al. (51)	Infusion control. Optimal levels of crystalloid infusion are 3.5ml-6ml/kg per hour to replenish lost fluids.	354	4.2%			7.3%				NA	0.8%	NA	4.1%
Khouri et al. (85)	Subcutaneous heparin was administered postoperatively. Less risk of microvascular thrombosis was noted.	493	NA	8.3%	NA	NA	NA	NA	2.7%	NA	4.1%	9.9%	6.25%
Eley et al. (86)	Vasopressor use. It is not recommended to administer vasopressors prior to dissection. Vasopressor administration after flap mobilization did not impact perfusion quality. It is not recommended to use epinephrine and dopexamine, as they correlate with a higher complication rate. Vasopressors are recommended postoperatively to improve overall perfusion quality	24	Perfusion change assessment with clinical application.										NA
	(combined with intravenous infusion).												
Enajat et al. (87)	Anticoagulant regiments evaluated. In total 325 mg of aspirin per os every 24 h or 5000 ME of LMW heparin subcutaneously every 24 h are methods of choice.	592	NA	3.4%	NA	NA	5.4%	NA	9.2%	NA	2.8%	2.6%	4.68%
Harris et al. (88)	Vasopressor use assessed. Dobutamin with norepinephrine combined therapy show positive effects in flap perfusion quality.	496	SM	5.2%	NA	NA	1.4%	SM	1.6%	NA	2.2%	NA	4.66%
Riva et al. (40)	Anithrombotic therapy regimens evaluated. Dextran is contraindicated and is harmful to postoperative flap stability. Increases complication rate. Antithrombotic therapy with dextran and/or PGE1 did not impact flap viability.	1,351	Pharmacological antithrombotic agent assessment.										NA

## Conclusion

Breast reconstruction using DIEP-flap based surgical intervention is a specific choice in order to foster the structural restoration of anterior chest wall anatomy after a mastectomy. This surgery represents an important stage in complete functional, emotional, psycho-social, and aesthetic patient rehabilitation. Implant-based reconstruction of breast results in the associated risks in perioperative period and complications such as infection, implant loss due to capsular contractures during postoperative periods. This can enhance the rate of failure of breast reconstruction by 30% and implant loss up to 4 to 18% among all the prosthetic breast reconstructions making the patients, surgeons hard to take appropriate decisions in order to eliminate these complications. Hence, a successful surgical reconstruction without complications is the priority of any surgeon. This study provides evidence-based recommendations on patient management in the perioperative period, and postoperative periods. These recommendations have a strong potential to improve clinical results when performing breast reconstruction with the DIEP-flap, and will serve as a basis for new prospective studies on topics covered in this study. Completing a series of patient management strategies may facilitate a high-quality surgery with minimal blood loss and minimal postoperative complications, which further correlates with a better patient rehabilitation and improved overall quality of life.

## Limitations and Strengths

This study described an in-depth review of existing associations between patient management and clinical characteristics, and associated postsurgical complications pertinent to the DIEP flap-based breast reconstruction. For the first time a large stratification and systematization of data was deciphered pertinent to the DIEP flap-based surgical intervention. The main limitation of this study is the lack of direct statistical comparison, mainly due to the largely differing reporting styles of the selected publications. Nonetheless, our review offers a valuable and important insight for the application of DIEP flap-based surgery to minimize the postsurgical complications in clinical practice.

## Author's Note

Breast reconstruction using DIEP-flap can be a significant surgical choice for the tissue restoration of anterior chest wall anatomy after a mastectomy. This systematic review deciphers the evidence-based recommendations on patient management in the perioperative period after DIEP flap-breast reconstruction.

## Data Availability Statement

The original contributions presented in the study are included in the article/supplementary material, further inquiries can be directed to the corresponding author/s.

## Author Contributions

KC, MS, NB, DS, YG, JLi, JZ, IR, OS, RF, JLiu, and PL: conceptualized the study. NB, KC, DS, YG, MS, and PL: performed the literature analysis and wrote the original manuscript draft. NB, PL, RF, and KC: revised, edited, and extended the final draft. All authors have reviewed and approved the manuscript before submission.

## Funding

This study was supported by the National Natural Science Foundation of China (No. 81703158).

## Conflict of Interest

The authors declare that the research was conducted in the absence of any commercial or financial relationships that could be construed as a potential conflict of interest.

## Publisher's Note

All claims expressed in this article are solely those of the authors and do not necessarily represent those of their affiliated organizations, or those of the publisher, the editors and the reviewers. Any product that may be evaluated in this article, or claim that may be made by its manufacturer, is not guaranteed or endorsed by the publisher.
